# Acceptable durability of split inner table graft for the reconstruction of a bone defect in pterional craniotomies: a case series

**DOI:** 10.3389/fsurg.2023.1213648

**Published:** 2023-07-14

**Authors:** Gi-Young Kweon, Jaechan Park, Wonsoo Son

**Affiliations:** Department of Neurosurgery, College of Medicine, Kyungpook National University, Daegu, Republic of Korea

**Keywords:** craniotomy, intracranial aneurysm, reconstruction, surgical technique, clipping

## Abstract

**Objective:**

During a pterional craniotomy, the bone defect was reconstructed by a surgical technique using an autogenous bone graft instead of commercially available alloplastic materials. The technical feasibility, durability of the grafted bone, and cosmetic outcome were all evaluated.

**Methods:**

After a pterional craniotomy was performed, the bone defect at the frontobasal burr hole and drilled sphenoid wing was reconstructed using an autogenous split inner table graft (1 cm × 2 cm) harvested from the craniotomy bone flap.

**Results:**

The bone reconstruction technique was successfully performed on nine patients with intracranial aneurysms. After 12–19 months from the surgery, a volumetry study using three-dimensional skull images reconstructed from computed tomography angiography showed a minimal decrease in the area of the split inner table graft due to bone resorption in six patients, which ranged from 5.7% to 14.8%. In the other three patients, the bone resorption was more substantial, ranging from 21.2% to 27.5%. However, in the three latter cases, the resorption was mainly limited to the posterior part of the split inner table graft covered by the temporalis muscle and did not affect the cosmetic outcomes. The resultant cosmetic outcomes for the nine patients were all favorable, with only a slight or no anterior temporal hollow.

**Conclusion:**

The proposed surgical technique using a split inner table graft harvested from the craniotomy bone flap seems viable for reconstructing the bone defect at the frontobasal burr hole and drilled sphenoid wing after a pterional craniotomy.

## Introduction

A pterional craniotomy, or fronto–temporo–sphenoidal craniotomy, is one of the most widely used neurosurgical approaches, which allows access to numerous surgical targets, such as anterior circulation aneurysms and tumorous lesions in the parasellar region (1, 2). It leaves a bone defect at the frontobasal burr hole and drilled sphenoid wing. To prevent a postoperative anterior temporal hollow, the bone defect requires reconstruction using alloplastic materials or an autogenous bone graft (3–6). Autogenous bone grafts are cost-effective innovations and can substitute commercially available alloplastic materials in the case of poor supply.

The authors reconstructed the bone defect at the frontobasal burr hole and the drilled sphenoid wing after a pterional craniotomy using an autogenous split inner table graft harvested from the craniotomy bone flap. Although this reconstruction technique using a split inner table graft has been applied to other surgical procedures requiring the reconstruction of a bone defect at the anterior skull base, orbital roof, mastoidectomy site, retrosigmoid/suboccipital region, and burr holes (7–12), it was rarely applied to cases of a pterional craniotomy. Thus, the technical feasibility in the case of a pterional craniotomy, the durability of the split inner table graft in the bone defect, and its effect on the postoperative anterior temporal hollow were all evaluated.

## Materials and methods

### Patient population and data collection

In 2019, pterional reconstruction using a split inner table graft in a pterional craniotomy was performed on nine patients at the authors’ institution. A conventional pterional craniotomy was performed on six patients with a ruptured aneurysm, and a mini-pterional craniotomy was performed on three patients with an unruptured aneurysm. The medical records and radiological data of the patients were reviewed to obtain relevant clinical and radiological information.

The first postoperative computed tomography angiography (CTA) was performed on postoperative day 1. The next CTA was performed 12–18 months after surgery. The volumetry study was performed using three-dimensional skull images reconstructed from the CTAs. PiViewSTAR™ (INFINITT Co., Ltd., Seoul, South Korea), an electronic picture-archiving and communication system, was used to measure the area of the split inner table graft based on manually outlining the region of interest in the lateral view of the reconstructed skull. Any change in the area of the split inner table graft due to bone resorption was calculated based on the difference in the area of the inner table graft as seen in the images obtained on postoperative day 1 and 12–18 months after surgery. The Oulu resorption score was also calculated and reported (13, 14).

Each patient underwent a cosmetic assessment at the follow-up clinic after more than 1 year from the surgery. The frontotemporal head was examined for anterior temporal hollow or any depression at the frontobasal burr hole site. This study was reviewed and approved by the ethics committee at the authors’ institution.

### Surgical technique

A scalp incision was made behind the hairline in a gentle C shape, starting at the midline and reaching the root of the zygoma. To minimize the atrophy of the temporalis muscle, dissection was performed inferiorly to superiorly from the temporal line (retrograde dissection) (15) using a periosteal dissector. The authors made every effort to minimize the use of monopolar cautery, and they made a myocutaneous flap by subperiosteal elevation of both the scalp and temporalis muscle together. A free bone flap was then created using two burr holes in the usual manner: one at the frontobasal burr hole and one just above and anterior to the root of the zygoma. A high-speed drill with a footplate attachment was used to create a frontotemporal craniotomy, and the sphenoid wing was drilled out as usual. The dimensions of the craniotomy can be decreased in cases of a mini-pterional craniotomy for unruptured aneurysms.

**Figure 1:**
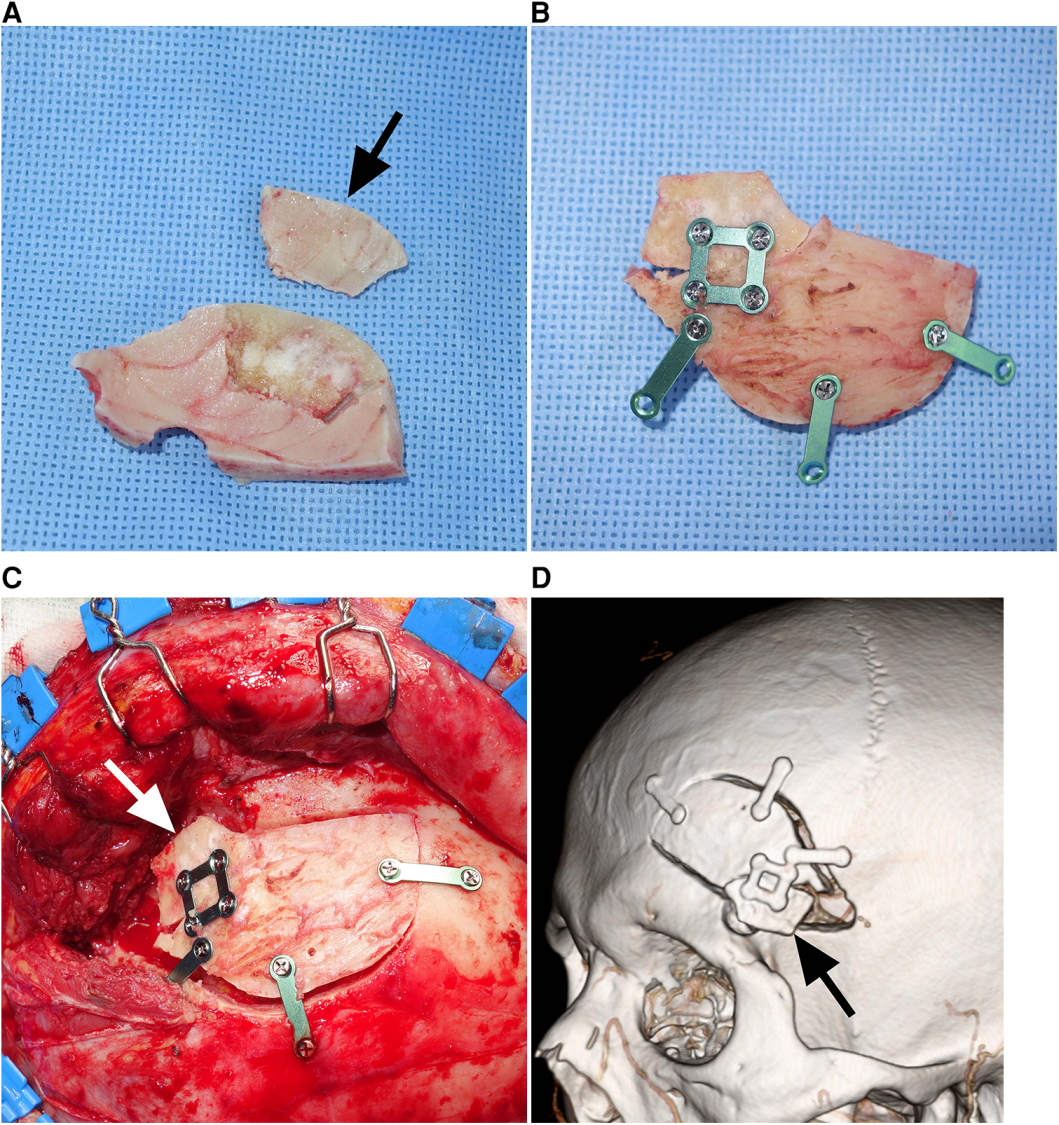
Reconstruction of a bone defect using a split inner table graft after a mini-pterional craniotomy (Patient 5). (**A**) Intraoperative photograph showing a Midas Rex C1 or equivalent drill bit cutting through the diploe of the bone flap. (**B**) Intraoperative photograph showing the bone flap and split inner table with dimensions of 1 cm × 2 cm (*arrow*). (**C**) Intraoperative photograph showing the inner table graft fixed along with the bone flap using a rectangular titanium plate. (**D**) Intraoperative photograph showing the split inner table graft (*arrow*) covering the frontobasal burr hole and drilled sphenoid wing.

Following aneurysm clipping and dural closure, the bone defect, including the frontobasal burr hole and drilled sphenoid wing, was reconstructed. A split inner table graft was harvested from the thickest part of the bone flap, the posteromedial part of the pterional bone flap (Figures 1A,B). The Midas Rex C1 or equivalent drill bit was used to cut through the diploe of the bone flap. A reciprocating saw could also be used to split the bone flap. A section of the inner table with dimensions of approximately 1 cm × 2 cm was used as the graft. The inner table graft was fixed along with the bone flap using a rectangular titanium plate to cover the bone defect at the frontobasal burr hole and sphenoid wing (Figure 1C). The inner table graft was trimmed to fit as close as possible to the size and contour of the bone defect to be covered. The whole bone flap was secured in place (Figure 1D). The temporalis muscle and scalp are sutured in separate layers.

## Results

### Technical feasibility

The reconstruction technique using a split inner table graft was attempted on a total of nine patients with a pterional craniotomy for clipping an intracranial aneurysm (Table 1), including both mini-pterional (*n* = 3) and conventional pterional (*n* = 6) craniotomies. The patient population consisted of four men and five women, with a mean age of 56.4 ± 10.8 years (range, 39–71 years).

**Table 1:** Clinical and radiological characteristics of nine patients who underwent pterional reconstruction using a split inner table graft.

Patient	Age (years), sex	Aneurysm location	Image follow-up interval (months)	Area of inner table graft (mm^2^), POD 1	Area of inner table graft (mm^2^), follow-up	Area change of inner table graft (%)	Oulu resorption score
1	67, F	AChA	14	105	99	−5.7	0
2	71, F	ACoA	18	151	139	−7.9	0
3	49, M	ACoA	14	117	100	−14.5	0
4	39, F	ACoA	14	151	141	−6.6	0
5	51, M	ACoA	12	241	212	−12.0	0
6	61, F	PCoA	12	189	161	−14.8	0
7	63, M	ACoA	12	160	117	−26.9	2
8	62, F	ACoA	15	137	108	−21.2	0
9	45, M	MCA	15	167	121	−27.5	2

AChA, anterior choroidal artery; ACoA, anterior communicating artery; MCA, middle cerebral artery; PCoA, posterior communicating artery; POD, postoperative day.

The inner table graft (1 cm × 2 cm) was split from the thickest part of the pterional bone flap. The splitting of the inner table graft and reconstruction of the bone defect were successfully performed in all patients. The additional surgical time required for the splitting and reconstructive procedures was negligible as the creation of the inner table graft and fixation to the bone flap were both performed by an assistant surgeon during the closure of the dura.

### Durability of the split inner table graft

The area of the split inner table graft immediately after surgery was measured using the first CTA performed on postoperative day 1. The reconstructed three-dimensional skull images demonstrated split inner table grafts with a mean area of 157.6 ± 40.3 mm^2^ (range, 105–241 mm^2^). Follow-up measurements of the inner table grafts were performed using the follow-up CTA, which was taken 12–19 months (mean ± SD: 14.8 ± 2.4 months) after the surgery. The mean area of the split inner table grafts based on the follow-up CTAs was 133.1 ± 36.0 mm^2^ (range, 99–212 mm^2^).

**Figure 2:**
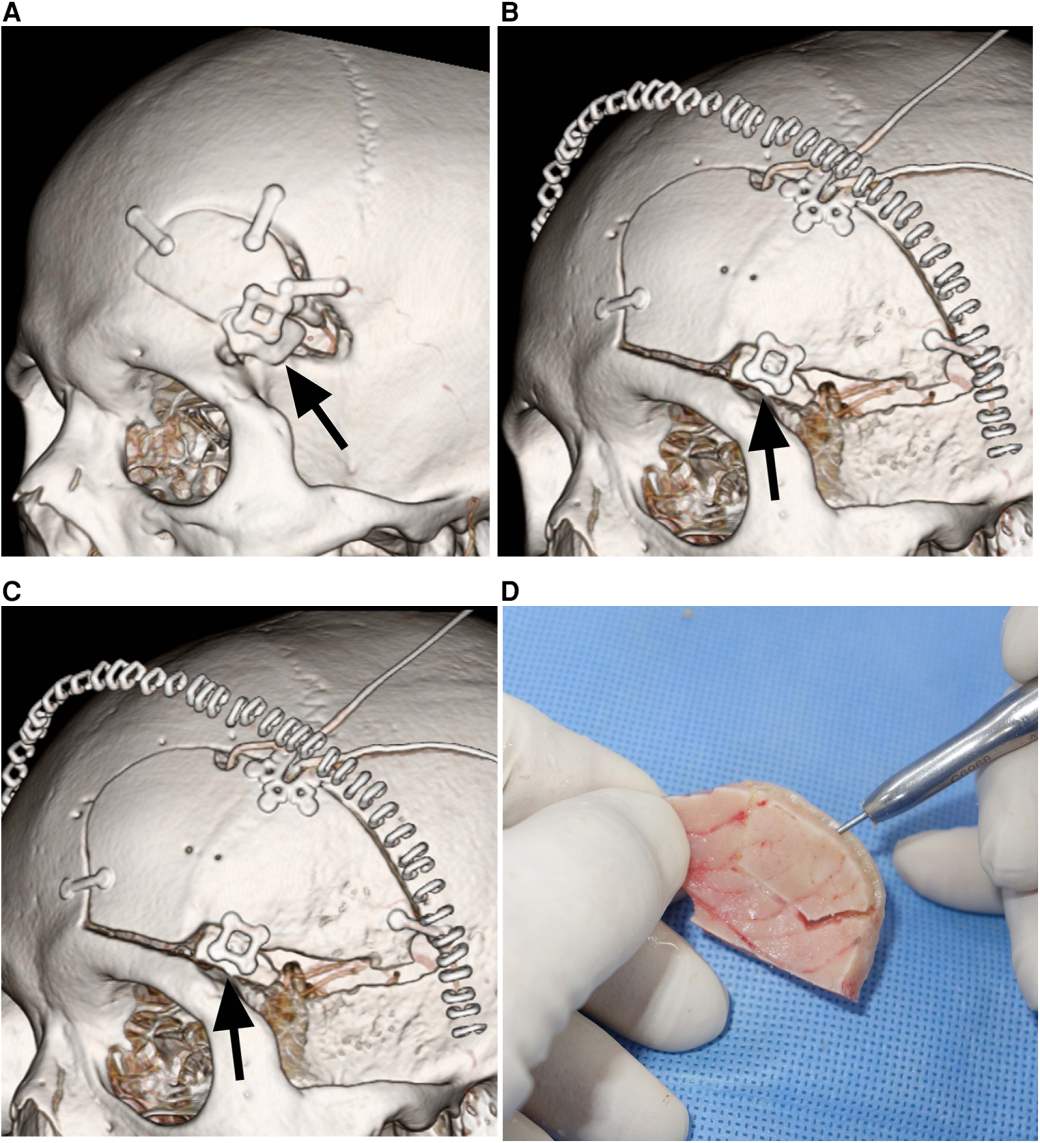
Durability of split inner table grafts. (**A**) Postoperative CT showing a mini-pterional craniotomy bone flap and the split inner table graft (*arrow*) for reconstruction in Patient 5. (**B**) CT 12 months after the craniotomy and reconstruction demonstrating the excellent durability of the split inner table graft (*arrow*) in Patient 5. (**C**) Postoperative CT showing a conventional pterional craniotomy bone flap and the split inner table graft (*arrow*) for reconstruction in Patient 7. (**D**) CT 12 months after the craniotomy and reconstruction showing 26.9% resorption of the split inner table graft (*arrow*) in Patient 7. The bone resorption is mainly limited to the posterior part of the inner table graft that is covered by the temporalis muscle.

The decrease in the area of the inner table graft due to bone resorption ranged from 5.7% to 27.5% (mean ± SD: 15.2% ± 8.3%), where six patients experienced a minimal decrease, ranging from 5.7% to 14.8% (Figures 2A,B), while the other three patients experienced a more substantial decrease, ranging from 21.2% to 27.5% (Figures 2C,D). In the case of the three patients with a more substantial decrease in the inner table graft, bone resorption mainly occurred in the posterior part of the inner table graft, which was covered by the temporalis muscle. There were no cases of infection of the grafted bone in this series.

### Cosmetic results

The operative site was examined during the follow-up clinic visit 12–18 months after surgery. No anterior temporal hollow was observed in seven patients, while a slight anterior temporal hollow due to temporalis atrophy was observed in two patients. None of the patients showed any focal depression at the frontobasal burr hole site.

The three patients with more substantial resorption of the grafted bone did not show an anterior temporal hollow or any cosmetic problems, as the bone resorption was limited to the posterior part of the grafted bone covered by the temporalis muscle.

## Discussion

Autogenous bone is used in reconstructing craniofacial defects as it provides the ideal structural and histocompatibility properties for osteointegration with a low risk of infection (16–18). In particular, the cranium, as a membranous bone, is preferred over endochondral bone due to its lower resorption rate, proximity to the surgical field in question, and minimal cosmetic or functional problems at the donor site (19–28). In animal experiments by Zins and Whitaker, the resorption rates of endochondral bone were as high as 65%–88%, whereas the resorption rates of membranous bone ranged from 17% to 20% (28).

The current surgical technique used a split inner table graft obtained from the bone flap removed during a craniotomy. This approach was chosen for the following reasons: (1) it was technically feasible without significant difficulty due to the requirement of a small graft size, with dimensions of approximately 1 cm × 2 cm; (2) there is no risk of donor-site morbidity, and (3) there is no risk of intracranial complications that can occur when splitting an outer table graft outside the craniotomy (29). This reconstructive technique of using a split inner table graft from a bone flap was already used by Kyoshima et al. in 10 cases, including the reconstruction of various bone defects produced by growing skull fractures, the removal of a tumor invading the skull, the excision of an intraorbital tumor, and a craniectomy (9). The postoperative course was satisfactory for all patients except one, who required repeated cranioplasty due to bone resorption.

No reports have been published on bone reconstruction in a pterional craniotomy using a split inner table graft. The durability of the grafted bone was the primary concern, as the split graft included the inner table and minimal cancellous bone. The results of this study demonstrated favorable or acceptable durability of a split inner table graft in a pterional craniotomy. Of all the patients, two-thirds showed minimal bone resorption of less than 15%, while one-third demonstrated acceptable bone resorption of approximately 25%. As the bone resorption of 25% was mainly limited to the posterior part of the grafted bone under the temporalis muscle, the cosmetic outcome was still favorable without any anterior temporal hollow.

As the process of bone grafting induces osteoconduction, osteoinduction, and osteogenesis, the grafted bone needs to be in close contact with adjacent vital bones to achieve successful bone grafting (30). Thus, for successful bone reconstruction in a pterional craniotomy, the split inner table graft must be placed in close contact with the frontotemporal base and the sphenoid wing rather than the craniotomy bone flap.

In cases of a large cranial defect, autologous bone, especially if drilled or split, has a high risk of resorption. Alloplastic materials are, therefore, preferred to autogenous bone grafts (31–33). In a comparative study by Jeyaraj (5), titanium mesh implants and split calvarial outer table grafts were compared with each other after the reconstruction of a large fronto–temporo–parietal cranial defect. The titanium mesh cranioplasty afforded more benefits, including a shorter operating time, ease of manipulation, absence of donor-site morbidity, and absence of graft resorption. In contrast, the cranioplasty using multiple split calvarial grafts was much more laborious and time-consuming, with only a limited quantity of calvarial grafts harvested.

With regard to a small bone defect due to a craniotomy, various alloplastic materials, including porous high-density polyethylene implants, hydroxyapatite and carbonated apatite cements, methylmethacrylate, and titanium mesh, have all been used (3, 29, 34–37). Recent studies report that ceramics are superior to polymers in terms of cosmetic and functional outcomes (38). However, split inner table grafts can also be used for small bone defects as they are biocompatible, inert, non-thermo-conductive, radio-transparent, rigid, inexpensive, and easily applied.

An anterior temporal hollow is a common sequela following a pterional craniotomy. It is caused by a combination of factors, including underlying bone defect, atrophy of the temporalis muscle, and reduction of the soft tissues (39). Careful dissection of the temporalis muscle is regarded as the most important factor in reducing the incidence of a temporal hollow. However, bone defects can also be considered an additional contributing factor. Thus, the reconstruction of a bone defect using alloplastic materials or autogenous bone grafts is required, and surgical techniques to minimize atrophy of the temporalis muscle are recommended to prevent an anterior temporal hollow (15, 40, 41). On the one hand, there have been several reports of alternative methods to avoid temporal hollowing. Rychen et al. reported on the safety and efficacy of supraorbital craniotomy or the sylvian keyhole approach, which can minimize damage to the temporalis muscle (42–44).

This report only covers a small series performed by a single surgeon. However, this is the first clinical report to use a split inner table graft for a pterional craniotomy, and the results advocate the feasibility of the proposed surgical technique and acceptable durability of a split inner table graft, thereby representing a viable option for pterional reconstruction. A larger prospective study is needed to prove the efficacy and safety of the proposed surgical technique.

## Conclusion

The proposed surgical technique using a split inner table graft harvested from the craniotomy bone flap seems viable for reconstructing the bone defect at the frontobasal burr hole and drilled sphenoid wing after a pterional craniotomy. The procedure was feasible without significant difficulty. The grafted inner table was durable with minimal or acceptable bone resorption. The resultant cosmetic outcomes were favorable, with slight or no anterior temporal hollow.

## Data Availability

The raw data supporting the conclusions of this article will be made available by the authors, without undue reservation.
